# Analyzing Groundwater Quality Data and Contamination Plumes with GWSDAT

**DOI:** 10.1111/gwat.12340

**Published:** 2015-05-13

**Authors:** Wayne R Jones, Michael J Spence, Matthijs Bonte

**Affiliations:** 2 Shell Global Solutions International B.V., HSE TechnologyLange Kleiweg 40, Rijswijk, The Netherlands

The Groundwater Spatiotemporal Data Analysis Tool (GWSDAT) is a user-friendly, open source software tool used to analyze and report trends in groundwater quality monitoring data. GWSDAT is based on the open source statistical programming language R and Microsoft Excel. GWSDAT’s primary use is for interrogation and interpretation of groundwater monitoring data derived from contaminated sites. It has specific functionality for analyzing dissolved-phase concentration and light nonaqueous phase liquid (LNAPL) thickness trends and spatiotemporal smoothing to delineate dynamic contamination plumes. The software can handle large data sets with multiple monitoring locations, variable sampling events, and differing chemical constituents. GWSDAT has been used extensively in the assessment of soil and groundwater conditions at numerous assets around the world, including retail and manufacturing sites. The following are key benefits and value-added features of GWSDAT:

- Improved data transparency to design and optimize groundwater monitoring or remediation programs;
- Early identification of new releases, migration pathways, the need for corrective action, and stable or declining trends that may aid in site closure determinations;
- Clarity on the relations between dissolved solute concentrations, LNAPL thicknesses, and groundwater elevation; and
- Rapid interpretation of complex data sets from large monitoring networks (e.g., refineries, terminals)

The main functionalities of GWSDAT include trend analyses, data smoothing, spatiotemporal smoothing, and determination of contamination plume characteristics. Groundwater quality observation data for individual monitoring points can be fitted to a linear or log linear regression model, where the significance of a trend can be determined by using the Mann-Kendall approach, which is widely used for trend detection in groundwater and surface water studies (Bouza-Deaño et al. 2008; Wahlin and Grimvall 2010). Groundwater concentration maps are developed by using a spatiotemporal solute concentration smoother derived from a penalized-splines nonparametric regression (Eilers and Marx 1996). The simultaneous statistical smoothing over space and time generally provides a more accurate, more consistent illustration of a contamination plume when compared against contour maps of individual sampling rounds (Cressie and Wikle 2011). In general, the spatiotemporally smoothed plume will be less biased by missing sampling rounds or missing data within sample rounds (Cressie and Wikle 2011). GWSDAT calculates a number of quantitative plume metrics by using the Ricker (2008) method describing the temporal plume behavior based on the contaminant mass of the plumes, average concentration, plume footprint area, and location of the center of mass. All of these are based on a spatial integration of the plume concentrations above a predefined concentration threshold. Further details on the input and output features of GSWDAT can be found in the supporting information (Appendixes S1 and S2). Additional information on each of the methodologies (including descriptions of the R packages used, but excluding the plume metrics, which are added in the latest version of GWSDAT) can be found in Jones et al. (2014).

## Software Availability

- Hardware requirements: Standard PC 1 GB RAM or more, 32-bit or 64-bit processor.
- System requirements: Microsoft Windows (XP or later)
- Software requirements: R version 3.0.0 (www.r-project.org) or higher and Microsoft Excel 2003 or higher.
- Program size: 13 MB
- Availability: Free from www.claire.co.uk/GWSDAT and http://www.api.org/GWSDAT

## Supporting Information

**Appendix S1**. GWSDAT Input

**Appendix S2**. Results and Reporting
