# Supplementary material for: Analyzing Groundwater Quality Data and Contamination Plumes with GWSDAT
Source: Ground Water. 2015 May 13;53(4):513–4. doi: 10.1111/gwat.12340 (PMC4682477; doi:10.1111/gwat.12340)
Supplement: Supplementary file 1 [file gwat0053-0513-sd1.doc]

**Supporting Information for**

Analyzing groundwater quality data and contamination plumes with GWSDAT

Wayne R Jones^1,*^, Michael J. Spence^2^, Matthijs Bonte^2^

^1^Shell Global Solutions (UK) Ltd, Statistics & Chemometrics, Brabazon House, Concord Business Park, Threapwood Road, Manchester, United Kingdom.

^2^Shell Global Solutions International B.V., HSE Technology, Lange Kleiweg 40, Rijswijk, The Netherlands

*Corresponding author: Wayne Jones, wayne.w.jones@shell.com, +44 (0) 161 499 4617.

**Appendix S1: GWSDAT input, output and reporting**

Data entry is via a Microsoft Excel input template (Figure S1), comprising three data input tables. Groundwater concentration data is entered for different constituents in different wells as a function of time. Input in this table can also comprise ‘non–detects’ (which can be specified at either the detection limit or half the detection limit); groundwater elevations (which can be used for drawing additional groundwater contours), and light non–aqueous phase liquid (LNAPL) thickness (which can be automatically replaced by groundwater concentrations for interpolation purposes: either the maximum groundwater concentration observed in the dataset, or concentration calculated effective aqueous solubility). The second table contains the wells coordinates and the third table can be used to specify the location of a basemap (in ArcGIS shapefile format).


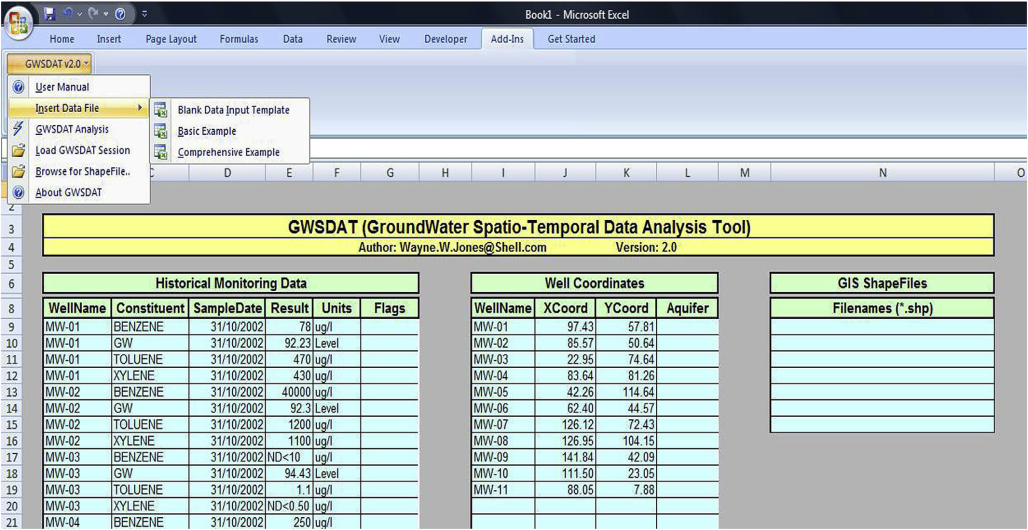


Figure S1. GWSDAT input sheet

**Appendix S2: Results and reporting**

The main output of GWSDAT consists of the screen shown in Figure S2 which has 4 panels (highlighted with A to D in Figure S2). In panel A, the user can select which constituent to plot, and whether to plot concentration trends, groundwater levels and/or LNAPL thickness. The user can also scroll through the time series and select a time slice (which is related to the concentration map and trend box, panels C and D, respectively). Panel B contains a graph with the observation data for the selected constituent and well (selected in panel A) and the selected trends (including 95% confidence percentiles). The grey line in the plot represents the time for which panels C and D are drawn.


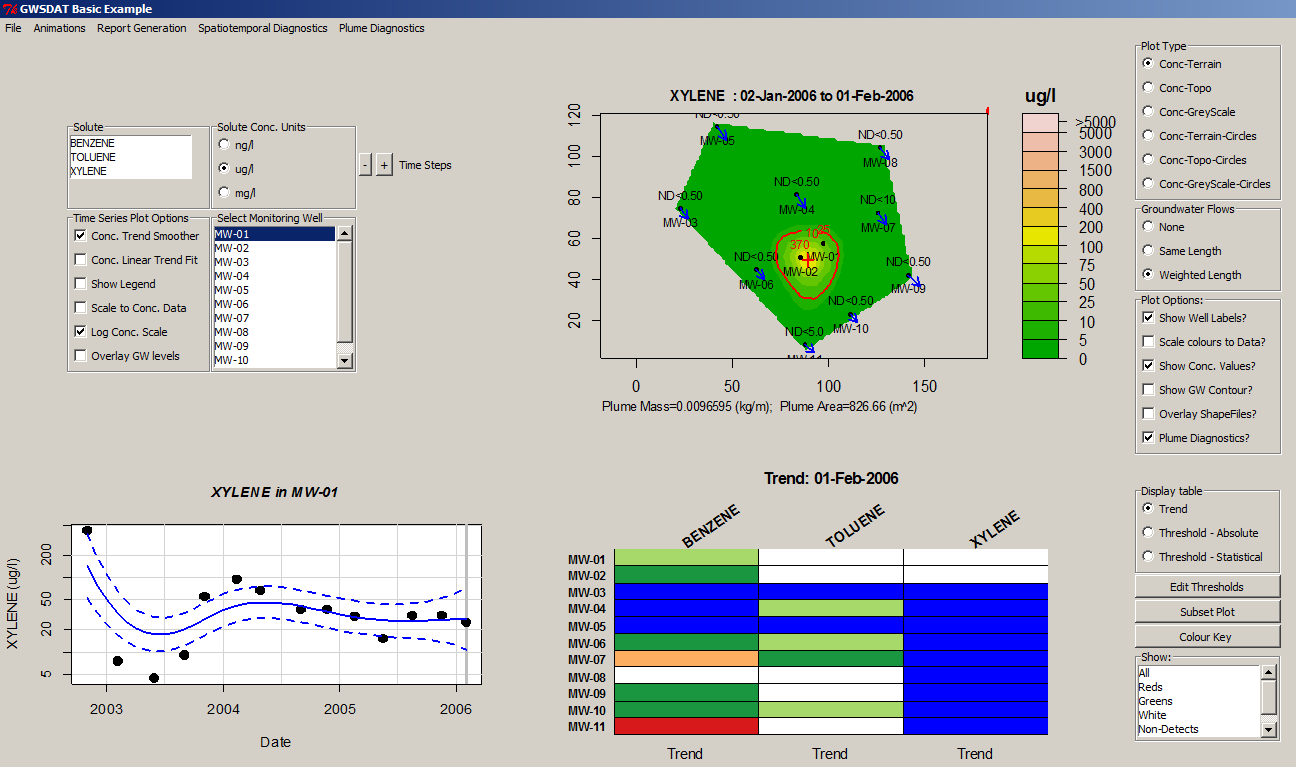


**D**

**A**

**C**

**B**

Figure S2. GWSDAT output sheet

The concentration map (panel C) shows a spatiotemporal model for a given time–slice (that was selected in panel A) including locations of wells and observed concentrations and with optional groundwater elevations and LNAPL thicknesses. The plume boundary contour is illustrated relative to a specified background or regulatory compliance concentration value. If the boundary contour is closed (i.e. the entire plume is captured by the spatiotemporal model), plume mass per meter aquifer thickness and plume area will be calculated. The trend and indicator threshold matrix (panel D) provides a fast way to determine concentration trends in the monitoring network for a given time slice (green = declining concentrations, white = stable, red = increasing). When using the matrix in ‘threshold mode’, the user can enter water quality threshold values and screen the data at the given time period against those thresholds (if the ‘statistical threshold’ option is used, the 95%–percentile of the data is screened against the threshold criteria).

GWSDAT can generate a number of different reports in different formats. The plots from the output screen can directly be exported as ‘jpeg’, ‘postscript’, ‘pdf’ files. It is also possible to export a sequence of plots capturing different time slices (both graphs from a single well, or spatiotemporal maps) and directly import these into Microsoft Word or Powerpoint. A complete series of graphs can be exported using the well reporting feature. This generates a matrix of graphs (one for each well) in which a selection of constituents can be plotted. Lastly, a number of summary graphs can be exported plotting the plume metrics based on the method by Ricker (2008) for any given constituent over time (Figure S3). This feature in particular, often in combination with a movie–like presentation of the contour plots in Powerpoint, rapidly creates a comprehensive view of plume behavior.


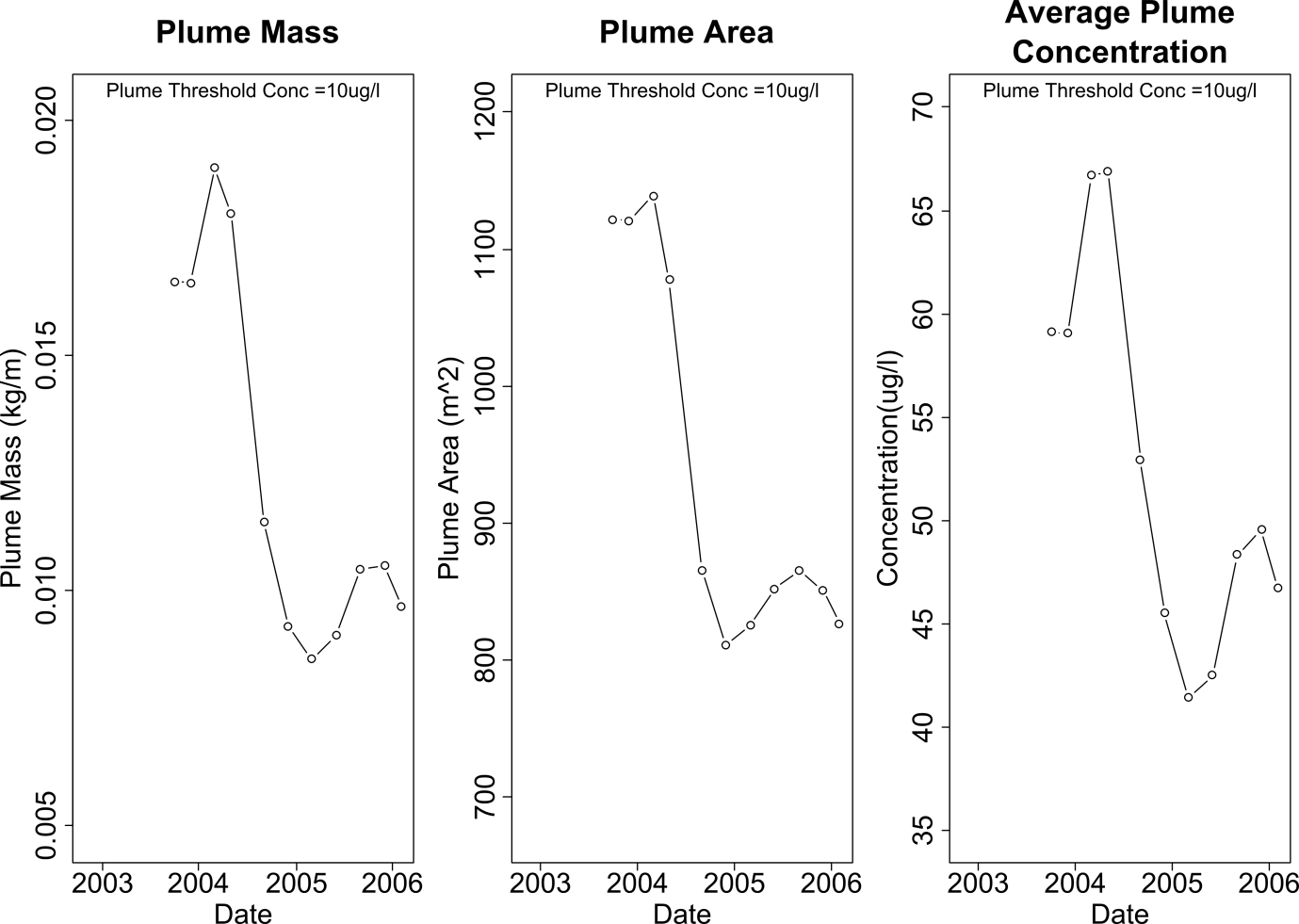


Figure S3. Example of the summary output of plume metrics: plume mass (left), plume area

**REFERENCES**

Ricker, J.A. 2008. A Practical Method to Evaluate Ground Water Contaminant Plume Stability. *Ground Water Monitoring & Remediation* **28,** no. 4: 85–94.
